# Information Concerning the American Society of Dental Surgeons

**Published:** 1841

**Authors:** Solyman Brown

**Affiliations:** Secretary.


### INFORMATION

### *Concerning the American Society of Dental Surgeons.*

—

[ BY SOLYMAN BROWN, M. D. SECRETARY. ]

—

Since the issue of the double number of this journal, containing extracts from the minutes of the first meeting of the Society, the Secretary has been repeatedly interrogated, both verbally and by letter, as to the prospects and designs of this extensive professional association in the United States. He has replied as fully and promptly as circumstances would permit, to all these inquiries, so far as he has been made acquainted with the views and intentions of the pricipal agents in the great work of organized reform in that department of surgical practice which falls exclusively to the lot of the surgeon dentist. But he has found it impossible to exhibit the subject in its various aspects, either in the form of correspondence by letter, or in occasional conversations ; and he has, therefore, consented to devote a few pages of the present number of the journal to this important topic.

For the purpose of imparting to the subject as much arrangement as the nature of the case will admit, it will be well to consider it under the two following heads :—*The objects to be attained—and the means of their attainment.*

Among the primary objects kept steadily in view by the projector of this society and his professional coadjutors, may be alledged—first :—THE PUBLIC GOOD, resulting from the united efforts of the most distinguished and enterprizing dental practitioners in the United States, aided by those of other countries, in settling the best methods of practice in all forms of dental disease.

Under this head of our subject, will be classed the effort to collect the scattered fragments of knowledge in relation to diseases of the teeth—their causes and remedies—into the form of a SCIENCE.

How much soever may be done by isolated individual effort to acquire personal distinction and emolument, benefitting a narrow circle of patients, the true philosopher and genuine philanthropist embraces within the scope of his vision a wider horizon. Leaping with benevolent dexterity from the circle of selfishness, he stands erect in the unlimited dominions of Truth and Charity, performing the appointed duties of his sublunary existence for the benefit of mankind. Not so with those who contract themselves like the tortoise within their own shells, and make no excursions except for booty.

In this remarkable age, where all the truly magnificent purposes of man are effected by combinations of individuals, acting with united effort for the production of grand results, it is really surprising to hear men of moderate pretensions to wisdom, talking of the superior advantages of private and individual exertions in the cause of dental science : and yet, perhaps, there always will be persons in every age, who, like the Sicilian mechanist and geometer, are waiting till they find a fulcrum upon which they can place a lever that will enable them by their own unassisted power to move the world.

A *second* object of the projectors of this society, is to embody all regular and worthy dental practitioners throughout the United States, in a properly organized fraternal association for the suppression of quackery and imposture.

It will not be denied that there has long been, and still is, abundant necessity for such a consummation. What profession was ever more disgraced by impudent pretenders, than that of Dentistry ? Medicine and Surgery have had some barriers against the inroads of those brainless barbarians who would mercilessly overrun the fair dominions of the healing art ; but in what part of our country was dentistry ever protected by law, or its charlatans resisted by combination ? Is it not time then, that all those honourable and capable men who have embraced this department of surgery as their profession for life, should unite to expel these barbarous Canaanites from their desecrated borders ?

To this end it will be the constant effort of the members of the American Society of Dental Surgeons to bring within their fraternity, all those deserving individuals in every section of our country, who are desirous of introducing true theory and honourable practice into the profession.

No individual who is worthy of its patronage, will be excluded from the fellowship of this society, provided the proper efforts be made, either by himself or his friends, to make known to the Executive Committee, the character of his claims.

It will be evident, on a moment's reflection, that the attainment of these important objects,—namely ; the arrangement of dental knowledge into scientific form, and the exclusion of quackery as far as practicable from the ranks of the profession, pre-supposes many minor objects of very great importance.

Among these is—first : A COMMUNITY OF KNOWLEDGE. Empyricism can effect but little in imposing on the credulity of the public without the aid of its secret nostrums and infallible specifics ; and while the public themselves allow more than one half the columns of every newspaper of our country to be filled with the advertisements of quack doctors and quack dentists, emblazoning in staring capitals the virtues of mysterious drugs and wonderful operations, to which it would be barbarous to subject the constitution of a dog, can it be reasonably expected that regular practitioners, in any department of the healing art, will be properly respected ? But, it will be asked, what is the remedy ? and we shall be told that mankind are so fond of being deceived by cunning and specious appearances that reason never will be their conductor. To this I answer, throw open wide the gates of public instruction. Labour to make knowledge as free and as universal as the atmosphere which we breathe. Send the school master still more generally abroad in the land; but more especially, let medical men and their public lecturers expose the folly and the madness of entrusting human life to the secret spells of alchemy and witchcraft. The truth is, the people must be enlightened on the subject of their physical organization, and the best modes of avoiding disease. The laws of health must be extensively promulgated. But in an especial manner, the professor of the healing art must make common stock of all the valuable facts which have been accumulated by experience, or unveiled by discovery. How unprofessional and malevolent would be the conduct of the selfish churl who could wish to conceal knowledge that might be useful to his race! The very fact of concealment should instantly deprive him of the confidence of mankind. And yet how frequently do we see the easily deluded multitude, thronging in herds the door of the empyric who pretends to possess some secret known only to himself! How much better would it be to base professional character upon a faithful application of known principles, than to labour to dupe the ignorant by the trickery of mysticism !

In the second place :--The encouragement of genuine merit and well intended effort in the cause of humanity, will have the effect of sustaining a respectable class of dental practitioners, to whom alone the public will, in time, learn to look for relief in dental diseases. Hitherto, community has been almost wholly unimformed as to the comparative competency of the several classes of professed surgeon dentists. This society will have power to establish lines of demarkation between the truly competent and the mere pretenders to dental knowledge. Such as deserve support will be sustained and encouraged by the combined influence of the entire dental association in the United States, insomuch that the simple fact of membership will constitute a passport to public favour.

But it must of necessity require some little time before the necessary information can be obtained by the Executive Committee, to enable them to recommend to the society, every individual in the profession, who may deserve its protection and support. But as soon as this object can be fully effected, those who have nothing to recommend them but the arts of quackery and the vices which should disgrace them, will be compelled to feed upon the gullibility of that portion of society who prefer ignorance to knowledge, and vice to virtue.

In the third place :—the elevation of the profession from the condition of scattered individuals to the rank of an organized association, recognized by the laws of the land, and acknowledged by similar bodies among the other professions, will be one of the earliest effects of the formation of the American Society of Dental Surgeons.

In the fourth place: — I may allege, with great propriety, *the cultivation of the social virtues*, and their legitimate pleasures, among the intelligent members of a widely extended profession, as by no means an unimportant effect of the organization of this society.

It will scarcely be doubted, much less denied, that objects like these are worthy of the most ardent desire, and the most vigorous prosecution. The *means*, therefore, for their attainment are well deserving our deliberate attention.

First—then, it may be observed, that annual meetings of the society will be holden in some of our larger cities, which it will be practicable for many of the members to attend, for the purpose of imparting and receiving knowledge necessary to professional eminence and usefulness. An interchange of views and opinions will be a distinguishing feature of these assemblies, and result in the greatest good to community at large.

Secondly:—At these annual meetings, written dissertations will be read by members previously appointed to that duty, on subjects of acknowledged interest to the profession. Besides these, an opening address is provided for by the By Laws, in addition to other sources of rational amusement and instruction.

Thirdly:—New associates will be annually admitted to the society, from among those younger members of the profession who have qualified themselves to stand in the places of those who have been removed from its ranks by death or otherwise. Indeed, the natural augmentation of the population of these States, from year to year, will demand large accessions to the number of dental operators, in order to keep pace with the wants of society.

Fourthly:—A system of popular Essays, in the form of small tracts, are in progress of active preparation, twelve of which are expected to be ready for publication and delivery by the time of the next annual meeting of the society. These brief essays, on the most important topics connected with the diseases of the teeth, are designed to be distributed by the members of the society among their patients. They are, therefore, intended to instruct, not so much the profession as the community at large, on the several subjects of which they treat. These tracts, it is hoped, will be issued at a cheap rate and in large quantities to meet the general demand.

Fifthly:—The subject of conferring the degree of dental Surgery, has engaged the early attention of the society, and the committee appointed to prepare the Diploma, and its appropriate seal, has already nearly completed its task. Every member of the society, honorary as well as active, will be entitled to a Diploma by paying into the treasury a stipulated sum, to be used for the benefit of the society. Although the simple act of conferring a scientific degree, does not of itself impart any additional knowledge, it may be made, at least, an evidence of knowledge already acquired by theory and confirmed by practice.

Sixthly:—The encouragement of Dental Colleges like that in successful operation at Baltimore, is a favourite object of this society, considered as one of the means of accomplishing the great objects of the association. Whether the society will organize a college of its own, or merely encourage the formation of several in various sections of the union, remains yet to be determined. There cannot exist a doubt, that the constant demand for properly educated dental practitioners in twenty-six states, besides several territories soon to become states, will require more than one dental college as soon as this mode of professional education shall be generally pursued. When we reflect that the necessities of community will require at least one dentist to every four physicians throughout the land, in order to counteract the luxurious habits, the hereditary tendencies, and the inveterate negligence of the inhabitants on the score of cleanliness of the teeth, we arrive at the necessary conclusion that schools of dental instruction will be demanded in the ratio of at least one to four, compared with those of general surgery and medicine.

Seventhly. The dental magazine already established, being the first publication of the kind in any age or country, so far as history informs us, has received the decided approbation of the Society, with the assurance of its constant support. Although the conductors and original projectors of the American Journal of Dental Science, were quite willing to resign the publication into the hands of the society, that body was disposed to decline the transfer, believing that the periodical would be best conducted on its present plan, modified perhaps, on the appearance of the second volume, so as to assume the form of a quarterly, instead of a monthly publication.

By the time of the second annual meeting of the Society, in August next, much progress will be made towards bringing the association into regular form, provided the Agent appointed to make application for a charter to the Legislature of the State of New York, at its ensuing Session, shall meet with desired success. Indeed, so few, and so reasonable are the powers and privileges requested in this charter, that the success of the application is scarcely problematical. By the period abovementioned, the Diploma will be ready for its seal, signature, and distribution. Twelve popular Essays, on very important topics, will be ready for distribution among the members, and, indeed, several of them will be put to press under the sanction and authority of the Publishing Committee before that period. Many additional members will be reported by the Executive Committee whose duty it is to examine the qualification and claims of all individuals of the profession proposed or applying for admission.

Moreover, the Examining Committee will be ready at the next meeting to investigate the qualifications of students from the offices of private instructors, who shall be presented for examination.

The accomplishment of all these important objects, in the compass of a single year, will require the faithful attention of the officers, committees and active members of the society during the few remaining months :—and it is confidently believed that after the next annual meeting the association will be in full and successful operation in all departments of its use.

Perhaps some of the readers of the Journal are ready to inquire :—" What are the essential qualifications for membership in the American Society of Dental Surgeons."

To this question the secretary most cheerfully responds, in conformity to the general sentiments freely expressed by the members in attendance at the time of the formation of the society, and the adoption of its constitution.

And first :—The original founders of the society were unanimous in the resolution to admit knowingly no individual as a member, with whom they could not cheerfully associate as a gentleman of unimpeached moral character.

In the dental profession, as in all others, there are men who scruple not to trample openly upon the received laws of order, morality and religion ; and inheriting as they do the contempt of mankind, it cannot be reasonably expected that gentlemen, to whom these laws are sacred, will open their arms to so foul an embrace. Such men, knowing as they must their own character, will spare themselves labor and disappointment by neglecting to present themselves at the doors of the society. They may rely upon it, that there are men within its walls so resolute and fearless, and so determined in regard to the character of their associates, that not even the jeopardy of their lives would deter them from doing all in their power consistently with honor, to exclude the base and the profligate from any association to which themselves may belong.

In the second place :—Unprofessional practices and notorious acts of quackery, will exclude their author from a seat in the society.

It is thus that the profession at large are determined to come to the aid of that portion of the community who have been unwittingly the dupes of shameless imposture. If the public will co-operate wisely with this society, there will be no difficulty in thoroughly excluding dental empiricism from the land. But as in the case of the other branches of the healing art, surgery and medicine, there will no doubt, always be found a sufficient number of patients who will adhere to the folly of preferring quacks to regular and honourable practitioners, because perhaps, their imaginations are prone to be delighted with the glaring fictions of those empirical advertisements with which the columns of almost every newspaper in the land are so largely graced.

But thus far I have spoken only of *disqualifications*, which has prepared the way for answering directly the question before us—" What are the essential qualifications for membership in the American Society of Dental Surgeons ?"

*Respectable talents :—creditable acquirements :—professional integrity ;—and a good moral character*. These four requisites are esteemed indispensable to a useful performance of the difficult and delicate duties of the dental surgeon; and all these, therefore, will be deemed essential to a fellowship in this association.

After these explanations of the objects of the society, I cannot, perhaps more properly conclude my remarks, than by expressing my confident hope that those members of the profession, in various sections of the United States, who have expressed their hostility to the American Society of Dental Surgeons, because they were neither invited to be present at its first meeting nor elected on that occasion as members, will think more rationally of the whole matter, and adopt the gentlemanly course pursued by many others, whose merits were not known to the individuals of the first convention, and rejoice that the doors of admission are to be guarded with unceasing watchfulness and care. Many persons of this latter character are providing themselves with the proper credentials for making known to the Executive Committee their just claims to fellowship; and even without this precaution on their part, many will be elected at the next annual meeting who were forgotten, overlooked, or unknown, at the last. In this manner it is hoped, from year to year, the society will augment in numbers, wisdom and usefulness, as long as the republic shall endure.

